# Supplementary material for: Heat Stress during Summer Attenuates Expression of the Hypothalamic Kisspeptin, an Upstream Regulator of the Hypothalamic–Pituitary–Gonadal Axis, in Domestic Sows
Source: Animals (Basel). 2022 Oct 28;12(21):2967. doi: 10.3390/ani12212967 (PMC9657376; doi:10.3390/ani12212967)
Supplement: Supplementary file 1 [file animals-12-02967-s001.zip › animals-1986119-supplementary.pdf]

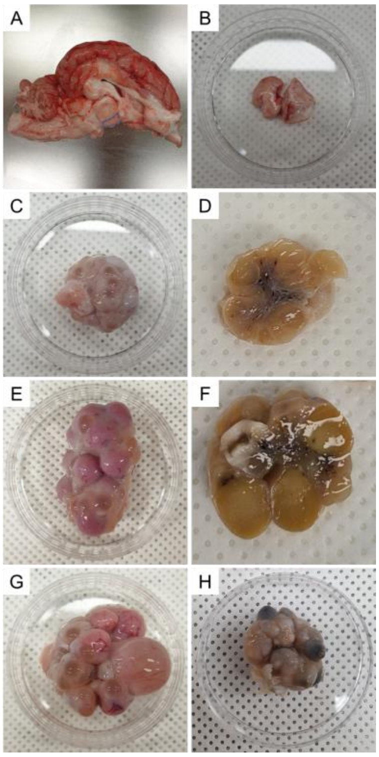

Supplementary Figure S1. Acquisition of samples from domestic sows. The hypothalamus (**A**), blue-dotted region) located at the undersurface of the brain lying just below the thalamus is bluntly isolated (**B**). The follicular phase (**C**,**D**) or luteal phase (**E**,**F**) are determined when growing follicles from small/medium follicles to Graafian follicles with regression of corpus luteum (CL) from previous cycles, or mature CL formation with growing small follicles but not too large follicles are observed, by morphological observation of the ovarian surface on gross (**C**,**E**) and cross-sectioned ovary (**D**,**F**). The subjects who showed cystic follicles (**G**) or corpus hemorrhagicum (**H**) in the ovary have been intensively excluded from the present study.

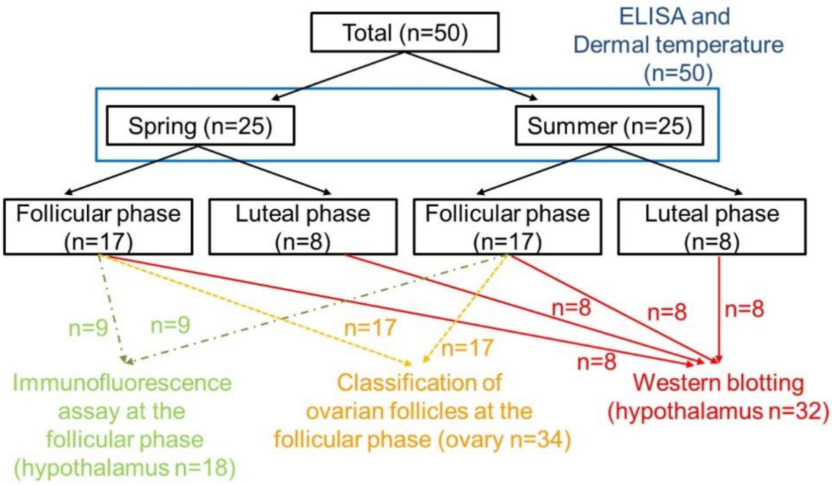

Supplementary Figure S2. Scheme of the number of samples to each assay.
